# Determination of Total Amino Acids in Infant Formulas, Adult Nutritionals, Dairy, and Cereal Matrixes by UHPLC–UV: Interlaboratory Validation Study, Final Action 2018.06

**DOI:** 10.1093/jaoacint/qsac083

**Published:** 2022-06-29

**Authors:** Greg Jaudzems, Christophe Fuerer

**Affiliations:** Nestlé Quality Assurance Center, 6625 Eiterman Rd, Dublin, OH 43017, USA; Société des Produits Nestlé SA, Nestlé Research, Route du Jorat 57, 1000 Lausanne 26, Switzerland

## Abstract

**Background:**

A method for the quantification of total amino acids (including taurine and excluding tryptophan) using ultra- HPLC separation coupled to UV detection (UHPLC–UV) was granted First Action status (AOAC **2018.06**) by the AOAC INTERNATIONAL Stakeholder Program for Infant Formula and Adult Nutritionals (SPIFAN) in 2018.

**Objective:**

An interlaboratory study was conducted to further assess method performance against the AOAC *Standard Method Performance Requirements* (AOAC SMPR^®^ 2014.013). Dairy and cereal matrixes were added to expand the scope of the method in collaboration with IDF (International Dairy Federation), ISO (International Organization for Standardization), and AACCI (American Association of Cereal Chemists International, now Cereals & Grains Association).

**Methods:**

Sixteen different matrixes were chosen to cover the requirements of AOAC, IDF/ISO, and AACCI. Blind duplicate samples were organized into specific series to ensure that each pair was analyzed on the same day. Fifteen laboratories returned results. Data from four laboratories were considered invalid and removed from the dataset. Remaining data were assessed according to the Appendix D of the AOAC *Official Methods of Analysis*^SM^ (guidelines for collaborative study procedures).

**Results:**

This method generally met the requirements listed in the SMPR for infant formulas and adult nutritionals, except for taurine. Method performance was comparable in dairy and cereal matrixes. Five different UHPLC instruments were used with either commercial or in-house reagents, demonstrating that the method is not limited to a single supplier.

**Conclusion:**

This method was recommended for Final Action in infant and adult/pediatric nutritional formulas by the AOAC SPIFAN Nutrients Expert Review Panel in April 2021, with the exception of taurine. The corresponding IDF/ISO Draft International Standard (DIS) was approved by national bodies in May 2022, and comments collected during the ballot were incorporated into this manuscript.

**Highlights:**

AOAC Official Method 2018.06 for the determination of total amino acids in infant formulas, adult nutritionals, dairy, and cereal matrixes was successfully validated in an interlaboratory study.

To date, there is no standard method for the determination of total amino acids in infant formulas and adult nutritionals. Total amino acid methods in other food and feed matrixes such as AOAC Method **994.12** (1), AOAC Method **985.28** (2), American Association of Cereal Chemists International, now Cereals & Grains Association (AACCI) 07–01.01 (3), and International Organization for Standardization (ISO) 13903:2005 (4) are all based on acid hydrolysis followed by ion exchange separation and post-column derivatization with ninhydrin, and analysis of sulfur amino acids requires an extra overnight oxidation step.

A faster method combining cysteine/cystine conversion by 3,3’-dithiodipropionic acid (DDP) into S-2-carboxyethylthiocysteine (XCys) during the acid hydrolysis step (5) and pre-column derivatization with 6-aminoquinolyl-*N*-hydroxysuccinimidyl carbamate (AQC) followed by reversed-phase ultra-HPLC (UHPLC) separation (6) was developed for the quantification of total amino acids and taurine (with the exception of tryptophan) in infant formulas and adult nutritionals (7). This method was granted First Action status by AOAC SPIFAN in September 2018 (AOAC Method **2018.06**), and a collaborative study was set up to provide additional method performance data. Dairy and cereal matrixes were added to the study to expand the scope of the method in collaboration with IDF, ISO, and AACCI (now Cereals & Grains Association).

## Experimental

### Interlaboratory Study Design

The interlaboratory protocol contained three phases: system qualification (to ensure baseline separation of all amino acids), laboratory qualification (using practice samples), and the interlaboratory study itself. For the last phase, the 32 blind duplicate samples were split into two different analytical series (four if needed) to ensure that sample pairs were analyzed within the same series. Results from the practice samples and interlaboratory study were communicated to the study director.

### Matrixes

Sixteen samples were categorized into SPIFAN, dairy, and cereal matrixes (Table 1). SPIFAN powder samples were first reconstituted in water as described in SMPR 2014.013 (8), and the other samples were analyzed as is.

**Table 1.** Description of the samples used in the interlaboratory validation

Sample	Blind code	ILS^a^ ID	Sample description^b^	Day	Reconstitution
SPIFAN samples					

S1	KGSZ273	4	NIST SRM 1869	1a	25 g + 200 g water, weigh out 220 mg
S1	LTCT316	5	NIST SRM 1869	1a	25 g + 200 g water, weigh out 220 mg
S2	KDOX966	1	IF, partially hydrolyzed, milk-based	1a	25 g + 200 g water, weigh out 220 mg
S2	ATAN351	6	IF, partially hydrolyzed, milk-based	1a	25 g + 200 g water, weigh out 220 mg
S3	SWUO667	7	IF, partially hydrolyzed, soy-based	1a	25 g + 200 g water, weigh out 220 mg
S3	MYHK654	3	IF, partially hydrolyzed, soy-based	1a	25 g + 200 g water, weigh out 220 mg
S4	ECHL425	8	IF, elemental, amino acid-based	1a	25 g + 200 g water, weigh out 220 mg
S4	UOPM297	2	IF, elemental, amino acid-based	1a	25 g + 200 g water, weigh out 220 mg
S5	XKIP216	26	IF, ready-to-feed, milk-based	2b	As is, weigh out 220 mg
S5	HYJU890	25	IF, ready-to-feed, milk-based	2b	As is, weigh out 220 mg
S6	CULF358	11	IF, milk-based	1b	25 g + 200 g water, weigh out 220 mg
S6	GBZC169	12	IF, milk-based	1b	25 g + 200 g water, weigh out 220 mg
S7	TJHR217	13	IF, soy-based	1b	25 g + 200 g water, weigh out 220 mg
S7	OACN211	9	IF, soy-based	1b	25 g + 200 g water, weigh out 220 mg
S8	EFXN778	10	Toddler formula, milk-based	1b	25 g + 200 g water, weigh out 220 mg
S8	BFAO941	15	Toddler formula, milk-based	1b	25 g + 200 g water, weigh out 220 mg
S9	LYNY751	14	Adult nutritional powder, low-fat	1b	25 g + 200 g water, weigh out 220 mg
S9	PZGP859	16	Adult nutritional powder, low-fat	1b	25 g + 200 g water, weigh out 220 mg

Dairy samples					

D1	DRGF365	19	muva M-0142—UHT skimmed milk	2a	As is, weigh out 220 mg
D1	QUET169	18	muva M-0142—UHT skimmed milk	2a	As is, weigh out 220 mg
D2	RJVA742	17	muva MO-0614—Whey powder	2a	As is, weigh out 50 mg
D2	BBCV185	24	muva MO-0614—Whey powder	2a	As is, weigh out 50 mg
D3	JNGK357	21	muva CA-0904—Sodium Caseinate	2a	As is, weigh out 50 mg
D3	JGMT273	22	muva CA-0904—Sodium Caseinate	2a	As is, weigh out 50 mg
D4	TKEH387	20	NIST SRM 1549a—Whole milk Powder	2a	As is, weigh out 50 mg
D4	ADKC392	23	NIST SRM 1549a—Whole milk Powder	2a	As is, weigh out 50 mg

Cereal samples					

C1	FMRM737	27	Bran pet food	2b	As is, weigh out 100 mg
C1	GHJR749	29	Bran pet food	2b	As is, weigh out 100 mg
C2	HWEU817	28	Dry pet food	2b	As is, weigh out 100 mg
C2	GXGS856	31	Dry pet food	2b	As is, weigh out 100 mg
C3	ZNDB409	30	NIST SRM 3233—Fortified breakfast cereal	2b	As is, weigh out 100 mg
C3	YWRU223	32	NIST SRM 3233—Fortified breakfast cereal	2b	As is, weigh out 100 mg

a ILS = Interlaboratory Study.

b SRM = Standard Reference Material; IF = infant formula; muva: muva kempten GmbH (Kempten, Germany).

### Participating Laboratories, Equipment, and Chemicals Used

Fifteen laboratories returned results. One laboratory did not analyze the two liquid samples and one laboratory only analyzed the SPIFAN powder samples. A survey was conducted among the participants to evaluate the diversity of instrument and reagents used during this study, and thirteen laboratories provided answers (see Supplemental Table 1).

Different instruments (six Waters Acquity, four Waters Acquity H-class, one Waters Acquity I-class, one Thermo 3000 RS, and one Agilent 1200 Infinity II) were used. All laboratories used the column specified in the method. For the mobile phase A, 11 laboratories prepared Eluent A from the AccQ·Tag Ultra Eluent A concentrate according to the protocol, one laboratory only used 120 mL of the concentrate (instead of 150 mL), and one laboratory used the Alternative Eluent A. For the mobile phase B, seven laboratories used the Eluent B from Waters and six laboratories used the Alternative Eluent B. Twelve laboratories used the AccQ·Tag Ultra derivatization kit from Waters and one laboratory used the Alternative Derivatizing Reagent.

## AOAC *Official Method*^SM^ 2018.06

**Total Amino Acids in Infant Formula and Adult**

**Nutritionals**

**UHPLC–UV**

**First Action 2018**

**Final Action 2021**

[Applicable for quantitative determination of total amino acids (AAs) including alanine, arginine, aspartic acid (combined with asparagine), cystine (dimer of cysteine, combined with cysteine), glutamic acid (combined with glutamine), glycine, histidine, isoleucine, leucine, lysine, methionine, phenylalanine, proline, serine, threonine, tyrosine, and valine in infant and adult/pediatric nutritional formulas. Method is not suitable for the determination of tryptophan and taurine.]

*Note:* Other matrixes such as dairy products, infant cereals, and pet foods were part of the multi-laboratory study for this method, but those data have not been evaluated for AOAC approval.

*Caution:* Refer to Material Safety Data Sheets prior to use of chemicals. Use appropriate personal protective equipment when performing testing. Because of the use of chemical solvents, acids, and reagents, perform sample preparation under a fume hood and take appropriate safety precautions.

### A. Principle

Proteins are hydrolyzed in 6M HCl for 24 h at 110°C in the presence of phenol, 3,3′-dithiodipropionic acid (DDP), and norvaline (Nva). Phenol is added to prevent halogenation of tyrosine. Nva is added as an internal standard. Cystine and cysteine are converted to S-2-carboxyethylthiocysteine (XCys) by DDP (5). After hydrolysis and neutralization, amino acids and XCys are derivatized with 6-aminoquinolyl-*N*-hydroxysuccinimidyl carbamate (AQC). Derivatized AAs are separated using reversed phase UHPLC with UV detection at 260 nm. (Fluorescence detection is also an option.)

During acid hydrolysis, glutamine (Gln) and asparagine (Asn) are converted to glutamic acid (Glu) and aspartic acid (Asp), respectively. Thus, Glu values represent the combined values of Glu and Gln, and Asp values represent the combined values of Asp and Asn. Cys values represent the combined values of cysteine and cystine since both are converted to XCys by DDP. Tryptophan is degraded by acid hydrolysis and cannot be analyzed by this method.

### B. Apparatus

- *UHPLC system.*—Any UHPLC system that sustains a pressure of approximately 62 MPa (9000 psi/620 bar) and can achieve baseline separation of the amino acids.
- *Chromatography column.*—ACQUITY UPLC™ BEH C18 Column, 130 Å, 1.7 μm, 2.1 mm × 150 mm (Waters, Product No. 186002353) or equivalent, provided baseline separation of the amino acids is achieved.
- *Adjustable micropipets.*—10, 20, 200, and 1000 μL and tips.
- *Vortex mixer.*
- *Analytical balance.*—Precision of 0.1 mg.
- *Heating block.*—55 ± 2°C.
- *Laboratory oven.*—110 ± 2°C.
- *Syringe filter.*—0.45 μm PVDF Millex^®^-HV (Millipore, Product. No. SLHV013NL) or equivalent
- *Syringes.*—2 mL.
- *Borosilicate glass tubes.*—10 mL (e.g., Pyrex) with screw cap.
- *Microtubes*.—1.5  and 2 mL.
- *Vial with screw cap*.—4 mL.
- *Glass* *screw-neck total recovery vial*.—12 × 32 mm (Waters, Product No. 186000384C or equivalent).

### C. Reagents

*Note*: Commercial references are only a guideline. Alternative chemicals or materials can be used provided their equivalence is demonstrated.

- *AccQ·Tag Ultra Derivatization Kit.*—Product No. 186003836 (Waters Corp.). Alternative derivatizing buffer: di-sodium tetraborate, decahydrate (CAS 1303-96-4). Alternative tagging reagent: 6-aminoquinolyl-*N*-hydroxysuccinimidyl carbamate (CAS 148757-94-2).
- *AccQ·Tag Ultra Eluent A concentrate*.—Product No. 186003838 (Waters Corp.). Alternative Eluent A: acetonitrile, gradient grade for liquid chromatography (CAS 75-05-8), formic acid (CAS 64-18-6), and ammonium formate (CAS 540-69-2).
- *AccQ·Tag Ultra Eluent B*.—Product No. 186003839 (Waters Corp.). Alternative Eluent B: acetonitrile, gradient grade for liquid chromatography (CAS 75-05-8) and formic acid (CAS 64-18-6).
- *Phenol.*—CAS 108-95-2.
- *3,3’-Dithiodipropionic acid (DDP).*—CAS 1119-62-6.
- *AA standard solution*.—Containing the following 17 AAs at 2.5 μmol/mL each (except l-cystine at 1.25 μmol/mL) in 0.1 mol/L HCl: l-alanine, l-arginine, l-aspartic acid, l-cystine, l-glutamic acid, l-glycine, l-histidine, l-isoleucine, l-leucine, l-lysine, l-methionine, l-phenylalanine, l-proline, l-serine, l-threonine, l-tyrosine, and l-valine.
- *l-* *Cystine.*—CAS 56-89-3.
- *Norvaline (Nva).*—CAS 6600-40-4.
- *NaOH* *pellets*.—Reagent grade (CAS 1310-72-2).
- *NaOH solution*.—1 M (CAS 1310-72-2).
- *NaOH solution (optional)*.—6 M (CAS 1310-72-2).
- *HCl,* *fuming 37% (12 M) GR for analysis.*—CAS 7647-01-0.
- *HCl*.—1 M (CAS 7647-01-0).
- *HCl*.—0.1 M (CAS 7647-01-0).
- *Laboratory water grade type 1.*

### D. Reagents and Standard Preparation

- *NaOH solutions*.—6, 0.2, and 0.05 M.
- *HCl solution*.—0.2 M.
- *1% (w/v) DDP in 0.2 M NaOH*.
- *0.1% (w/v) phenol in 12 M HCl*.
- *AccQ·Tag Ultra Derivatization Kit*.—Prepare the reagents included in the kit following the manufacturer’s instructions.
  1. *AccQ·Tag Ultra Borate buffer (reagent 1)*.—Ready-to-use solution. Alternative reagent: 5% (w/v) sodium tetraborate in water.
  2. *AccQ·Tag Ultra reagent (vials 2A and 2B)*.—Reconstitute AccQ·Tag Ultra reagent (vial 2A) according to the manufacturer’s instructions: Preheat a heating block to 55°C. Tap vial 2A lightly before opening to ensure all AccQ·Tag Ultra reagent powder is at the bottom of the vial. Rinse a clean micropipet by drawing and discarding 1 mL AccQ·Tag Ultra reagent diluent from vial 2B (ready-to-use solution); repeat twice. Draw 1.0 mL from vial 2B, transfer to the AccQ·Tag Ultra reagent powder in vial 2A, and cap vial tightly. Mix on a vortex mixer for approximately 10 s, and then heat vial 2A on top of the preheated heating block until the AccQ·Tag Ultra reagent powder is dissolved. Do not heat the reagent for longer than 10 min.
    Once reconstituted, the AccQ·Tag Ultra reagent is approximately 10 mM. Store reconstituted AccQ·Tag Ultra reagent in a desiccator at room temperature for up to 1 week.
    (*Caution:* AccQ·Tag Ultra reagent reacts with atmospheric moisture. Seal the container tightly when not in use. Do not refrigerate. Do not use discolored reagent, especially if yellow or green.)
*Alternative reagent.*—Into a 4 mL vial, weigh out approximately 3.0–4.0 mg AQC. Rinse a clean micropipet by drawing and discarding 1 mL acetonitrile; repeat twice. Add 1.0 mL acetonitrile to the AQC tube and cap vial tightly. Mix on a vortex mixer for approximately 10 s, and then heat vial on top of the preheated heating block until the AQC powder is dissolved. Do not heat the reagent for longer than 10 min.
- *Nva internal standards*.
  1. *10 mM Nva stock solution*.—Weigh out 117.16 mg Nva into a 100 mL volumetric flask and dilute to the mark with 0.1 M HCl.
  2. *2.5 mM Nva solution.—*Pipet 2.5 mL 10 mM Nva stock solution into a 10 mL volumetric flask and dilute to the mark with 0.1 M HCl.
    Store both Nva solutions at –20°C for up to 6 months as 2 mL aliquots.
- *Cystine calibration standards*.
  1. *10 mM cystine stock solution*.—Weigh out 240 mg cystine into a 100 mL volumetric flask and dilute to the mark with 0.05 M NaOH. Store 10 mM cystine stock solution at –20°C for up to 3 months as 1 mL aliquots.
  2. *1 mM cystine solution*.—Add 900 μL 0.05 M NaOH to 100 μL 10 mM cystine stock solution.
    The 1 mM cystine solution is freshly prepared for each analysis.
- *AA calibration standards (excluding cystine)*.
  1. *2.5 mM AA stock solution*.—AA standard solution is ready to use and contains 2.5 mM of each AA. [Although present in this solution, cystine is not used for quantification and is prepared separately; see section **D(g)**.] Store 2.5 mM calibration standard stock solution at –20°C for up to 6 months as 250 μL aliquots.
  2. *0.5 mM AA solution*.—Add 600 μL 0.1 M HCl to 150 μL 2.5 mM AA solution.
  3. *0.05 mM AA solution*.—Add 900 μL 0.1 M HCl to 100 μL 0.5 mM AA solution.
    Both 0.5 and 0.05 mM AA solutions are freshly prepared for each analysis.
- *Chromatography solvents (mobile phases)*.
  1. *Eluent A (Solvent A)*.—Prepare Eluent A from AccQ·Tag Ultra Eluent A concentrate as follows: Measure 850 mL water into a 1 L graduated cylinder. In a separate graduated cylinder, measure 150 mL AccQ·Tag Ultra Eluent A concentrate. Then, add the concentrate to the water and mix thoroughly.
*Note*: Eluent A concentrate, once opened, must be stored tightly capped at around 4°C. Dilute Eluent A is stable for 1 week at room temperature.
*Alternative Eluent A concentrate*.—840 mL 200 mM ammonium formate in water (12.61 g to 1 L water), 50 mL formic acid, and 110 mL acetonitrile. Prepare Alternative Eluent A from the concentrate as described in **D(i)**(*1*).
  2. *Eluent B (Solvent B)*.—AccQ·Tag Eluent B is supplied as a working solution; no additional preparation is required. Eluent B, once opened, must be stored tightly capped at around 4°C for no longer than 1 month.
*Alternative Eluent B*.—Use HPLC grade acetonitrile supplemented with 2% (w/w) formic acid (13.2 mL formic acid added to 1 L acetonitrile).
- *Wash solvents*.—The weak needle-wash solvent is 5% (v/v) acetonitrile in water. The strong needle-wash solvent is 95% (v/v) acetonitrile in water. The seal-wash solvent is 50% (v/v) acetonitrile in water.

### E. Sample Analysis

- *Sample preparation*.—
  1. *Infant formulas, adult nutritionals, ready-to-feed (RTF) liquids and liquid dairy samples.*—Reconstitute powders by adding 25 g powder to 200 g water and mix thoroughly. Weigh out 220 ± 20 mg reconstituted powders or liquids into a 10 mL glass tube with a screw cap. Report the sample mass to 0.1 mg. Complete to 1100 mg with water.
    To each tube, add 600 μL DDP solution (1% DDP in 0.2 M NaOH), 600 μL 0.2 M HCl, 200 μL 10 mM Nva stock solution (10 pmol/μL final concentration after derivatization), and 2500 μL phenol–HCl solution (0.1% phenol in 12 M HCl).
*Note*: Phenol–HCl solution has to be added under the fume hood.
  2. *Dairy powder samples.*—Weigh out 50 ± 5mg dairy powder samples into a 10 mL glass tube with a screw cap. Report the sample mass to 0.1 mg. Complete to 800 mg with water.
    To each tube, add 600 μL DDP solution (1% DDP in 0.2 M NaOH), 600 μL 0.2 M HCl, 500 μL 10 mM Nva stock solution (10 pmol/μL final concentration after derivatization), and 2500 μL phenol–HCl solution (0.1% phenol in 12 M HCl).
*Note*: Phenol–HCl solution has to be added under the fume hood.
  3. *Cereal powder samples.*—Weigh out 100 ± 10 mg cereal powder samples into a 10 mL glass tube with a screw cap. Report the sample mass to 0.1 mg. Complete to 1100 mg with water.
    To each tube, add 600 μL DDP solution (1% DDP in 0.2 M NaOH), 600 μL 0.2 M HCl, 200 μL 10 mM Nva stock solution (10 pmol/μL final concentration after derivatization), and 2500 μL phenol–HCl solution (0.1% phenol in 12 M HCl).
*Note*: Phenol–HCl solution has to be added under the fume hood.
    [For all samples prepared above as described in **E(a)**(*1*)–(*3*).]
    Sparge the tube for a minimum of 5 s with a stream of nitrogen to displace oxygen.
    Close tubes with screw caps and mix on a vortex mixer.
*Note*: Make sure the caps are perfectly clean (i.e., devoid of any particles) to ensure tightness and avoid evaporation during hydrolysis.
- *Cystine calibration standards preparation*.—Table **2018.06A** describes how to prepare calibration standards for converted cystine at 0–10 pmol/μL and Nva at 10 pmol/μL (all are final concentrations after derivatization).
*Note*: Phenol–HCl solution has to be added under the fume hood.
  Sparge the tube for a minimum of 5 s with a stream of nitrogen to displace oxygen.
  Close tubes with screw caps and mix on a vortex mixer.
*Note*: Make sure the caps are perfectly clean (i.e., devoid of any particles) to ensure tightness and avoid evaporation during hydrolysis.
- *Hydrolysis (of samples and cystine standards)*.—Place tubes in an oven at 110 ± 2°C for 24 ± 0.5 h.
- *Neutralization and dilution (of samples and cystine standards)*.—Take the tubes out of the oven. Allow hydrolysates to cool down and particles to settle prior to taking an aliquot. When transferring aliquots, pipet about 1 cm below the top of the liquid. Perform neutralization under the fume hood.
  1. *Reconstituted infant formulas,* *RTF* *liquid samples, dairy liquid samples, cereal powder samples, and converted cystine standards)*.—Transfer 0.2 mL of each hydrolysate into a 1.5 mL microtube, add 0.2 mL 6 M NaOH, and then 0.4 mL 0.1 M HCl. Mix well and filter through a 0.45 μm membrane filter into another 1.5 mL microtube.
  2. *Dairy powder samples*.—Transfer 0.2 mL of each hydrolysate into a 1.5 mL microtube, add 0.2 mL 6 M NaOH, and then 1.6 mL 0.1 M HCl. Mix well and filter through a 0.45 μm membrane filter into another 1.5 mL microtube.
- *AAs calibration standards preparation*.—Acid hydrolysis is not necessary.
Table **2018.06B** shows how to prepare 0.5 mL calibration standards at 0–25 pmol/μL and Nva at 10 pmol/μL (all are final concentrations after derivatization).
  The AA solutions are stable for 1 week when stored at 4 ± 2°C.
- *Derivatization (of samples, cystine standards, and AAs standards)*.—Derivatization converts free AAs into highly stable derivatives. Standards and samples are derivatized following the manufacturer’s instructions as described below.
  1. Preheat a heating block to 55°C.
  2. With a micropipet, add 70 μL AccQ·Tag Ultra Borate buffer [reagent 1, *see***D(e)**(*1*)] to a clean 12 × 32 mm glass screw-neck total recovery vial.
  3. Add 10 μL calibration standard, **E(e)**, neutralized sample solution, **E(d)**, or neutralized converted cystine standard, **E(d)**, to the vial.
  4. Mix briefly on a vortex mixer.
  5. Add 20 μL reconstituted AccQ·Tag Ultra reagent, **D(e)**(*2*), to the sample vial.
  6. Mix the solution immediately by pipetting up and down several times. Cap, mix on a vortex mixer immediately for several seconds, and tap the vial to ensure that no bubble is trapped.
  7. Let stand for 1 min at room temperature.
  8. Heat the vial in a heating block for 10 min at 55 ± 1°C.
- *UHPLC separation*.—
  1. Prime solvent lines for 5 min.
  2. Prime wash/sample syringes for four cycles.
  3. Allow the chromatographic system to stabilize before injecting standards and samples. Make sure the system pressure and initial conditions are stable before performing injections (around 62 MPa/9000 psi/620 bar).
  4. Before starting a series of analyses, inject two blanks (water) to condition the column.
  5. Inject 1 μL of each derivatized calibration standard and then inject 1 μL derivatized sample solutions. Perform single injections. Add a blank injection (water) at the end of each calibration series.
  6. Perform UHPLC under the conditions in Table **2018.06C**.
    Operating conditions may vary depending on the apparatus. Follow the supplier’s instructions. Examples of wash solvents used with Waters UPLC systems are given in section **D(j)**.
- *Peak identification and integration*.—Identify the AA peaks in the sample solution by comparison with the retention times of the corresponding peaks obtained in the calibration standards. If a peak has not been integrated correctly, call the recorded data and reintegrate.

To verify that the system is stable, inject a mid-level standard a minimum of three times (five times for U.S. Pharmacopeia requirements) and ensure that response and retention times have an RSD <2%.

Check that peaks are separated with a good resolution (baseline separation). If this is not the case, adapt the chromatographic conditions (e.g., gradient, temperature, tubing length, etc.) accordingly.

Verify that the derivatization reagent was present in excess. Excess reagent hydrolyzes to yield 6-aminoquinoline (AMQ), a noninterfering byproduct present on the chromatogram as the large peak first to elute. In samples, the response of the AMQ peak should not be smaller than that observed in the 25 pmol/μL standard, or the reaction and sample should be flagged and discarded. The derivatization peak at approximately 17 min prior to lysine can be ignored.

### F. Calculation and Expression of Results

- *Calibration curve*.—Establish the calibration curve from the six different calibration standards for each AA and converted cystine at the beginning of each series of analyses by plotting the response [peak area ratio of analyte (A_s_) versus internal standard (A_is_), multiplied by the concentration of the internal standard (C_is_); see below] against analyte concentration. Both concentrations are expressed in picomoles per microliter.
  $$\mathrm{Response}=\;\frac{A_{s}}{A_{is}}\times C_{is}$$
  Force the linear regression through zero.
  Check the linearity of the calibration (the correlation coefficient *R*^2^ must be above 0.99).
- *AA calculation*.—Calculate the amount of individual AAs present in the sample in picomoles per microliter from the calibration curve using the following equation:
  $$C_{s}=\frac{A_{s}\times C_{is}}{A_{is}\times S}$$
  where C_*s*_ = concentration of individual AA in the test sample solution in picomoles per microliter; A_*s*_ = peak area of individual AA in the test sample solution; C_*is*_ = concentration of internal standard injected in picomoles per microliter; A_*is*_ = peak area of internal standard chromatogram; and S = slope of the calibration curve (all curves are forced through zero; equation y = ax).
  Calculate the mass fraction, w, of each AA, in mg/100 g product, using the following equation:
  $$w=\frac{C_{s}\times M_{A}\times V_{s}\times d_{1}\times d_{2}}{m_{s}\times 10}$$
  where M_A_ = molar mass of individual AAs in grams per mole (*see*Table **2018.06D**); V_s_ = volume of hydrolysis solution in milliliters (typically 5 mL); d_1_ = dilution factor in the neutralization step, **E(d)** (typically 4 or 10); d_2_ = dilution factor in the derivatization step, **E(f)** (typically 10); m_s_ = mass of the test portion in milligrams; and 10 = combined factor to convert picograms to milligrams (10^−9^), milliliters to microliters (10^3^), and micrograms to 100 g (1/10^−5^).

**Table 2018.06A.** Cystine concentration (final, after derivatization)

Solution	10 pmol/µL	5 pmol/µL	2.5 pmol/µL	1 pmol/µL	0.5 pmol/µL	0 pmol/µL
Cystine solution	200 µL^a^	100 µL^a^	50 µL^a^	200 µL^b^	100 µL^b^	0 µL
Water	900 µL	1000 µL	1050 µL	900 µL	1000 µL	1100 µL
1% DDP in 0.2 M NaOH	600 µL	600 µL	600 µL	600 µL	600 µL	600 µL
0.2 M HCl	600 µL	600 µL	600 µL	600 µL	600 µL	600 µL
10 mM Nva stock solution	200 µL	200 µL	200 µL	200 µL	200 µL	200 µL
0.1% phenol in 12 M HCl	2500 µL	2500 µL	2500 µL	2500 µL	2500 µL	2500 µL

a 10 mM cystine stock solution.

b 1 mM cystine solution.

**Table 2018.06B.** Amino acid concentrations (each, final, after derivatization)

Solution	25 pmol/µL	10 pmol/µL	5 pmol/µL	1 pmol/µL	0.5 pmol/µL	0 pmol/µL
Amino acid solution	50 µL^a^	100 µL^b^	50 µL^b^	100 µL^c^	50 µL^c^	0 µL
Water	50µL	100µL	50µL	100µL	50µL	0 µL
2.5 mM Nva in 0.1 M HCl	20 µL	20 µL	20 µL	20 µL	20 µL	20 µL
0.1 M HCl	380 µL	280 µL	380 µL	280 µL	380 µL	480 µL

a 2.5 mM AA stock solution.

b 0.5 mM AA solution.

c 0.05 mM AA solution.

**Table 2018.06C.** UHPLC conditions

Column temp, °C	50			
UV detector, nm	260			
Injection volume, μL	1			
Flow rate, mL/min	0.4			
Mobile phase A	Eluent A			
Mobile phase B	Eluent B			

Elution gradient	Time, min	A, %	B, %	Curve

	0.00	99.9	0.1	
	5.50	99.9	0.1	2
	15.22	90.9	9.1	7
	20.47	78.8	21.2	6
	21.26	40.4	59.6	6
	21.29	10	90	6
	22.84	10	90	6
	26.00	99.9	0.1	6
	32.00	99.9	0.1	6

**Table 2018.06D.** Molar masses (M_A_) of amino acids (g/mol)

Aspartic acid	133.11
Threonine	119.12
Serine	105.09
Glutamic acid	147.13
Proline	115.13
Glycine	75.07
Alanine	89.10
Cystine	240.30
Valine	117.15
Methionine	149.21
Isoleucine	131.18
Leucine	131.18
Tyrosine	181.19
Phenylalanine	165.19
Lysine	146.19
Histidine	155.16
Arginine	174.20

## Results and Discussion

### Laboratory Qualification

Fifteen laboratories returned data. Eight laboratories submitted data from the practice samples before proceeding to the interlaboratory study. All eight laboratories provided satisfactory results (i.e., at least 30 out of their 35 reported values were within 20% of the single-laboratory validation average value) and qualified to participate to the interlaboratory test (Table 2). The remaining seven laboratories directly proceeded to the interlaboratory test phase and returned the results. All results are reported in Supplemental File 1.

**Table 2.** List of participating laboratories detailing the results of their qualifying round and the overall results of the interlaboratory study^a^

Lab															
	1	2	3	4	5	6	7	8	9	10	11	12	13	14	15
Qualification round															

Values within norm	33/35	DNR^b^	DNR	31/35	DNR	DNR	34/35	35/35	DNR	34/35	DNR	34/35	DNR	31/35	33/35
Qualification	Yes	NA^c^	NA	Yes	NA	NA	Yes	Yes	NA	Yes	NA	Yes	NA	Yes	Yes

Interlaboratory test															

Values returned	558	558	558	558	558	558	490	558	558	276	490	557	558	558	558
Average	2.02	0.95	0.98	0.99	0.62	0.97	0.89	0.93	0.96	0.94	0.96	0.98	0.84	0.96	0.95
SD	0.81	0.12	0.08	0.13	0.10	0.09	0.37	0.08	0.09	0.06	0.10	0.16	0.15	0.11	0.11
RSD	40%	12%	9%	13%	17%	9%	41%	8%	9%	6%	10%	16%	18%	11%	11%

*z*-score (average)	18.70	−0.21	0.26	0.37	−5.67	0.03	−0.75	−0.47	−0.01	−0.24	0.11	0.43	−2.12	−0.08	−0.12
|*z*|>2, %^d^	74	15	4	4	91	2	44	0	4	1	3	5	46	8	6

a The average, SD, and RSD were calculated from individual amino acid values normalized to the average value of each analyte/sample pair.

b DNR = Did not return (results from the qualification round).

c NA = Not applicable.

d Proportion of z-score values exceeding 2 standard deviations.

### Data Validation

The data set was first analyzed to detect laboratories that should be excluded from the study because of a global bias or extensive spread. To do so, mean values were first calculated for each analyte/sample pair. Then, all individual amino acid values were expressed relative to the calculated mean. Finally, the average relative value and standard deviation was calculated for each laboratory. As shown in Table 2, Laboratories 1, 5, and 13 showed a global bias (their average value ± SD did not include the calculated mean), and laboratories 1 and 7 showed an extensive spread (their RSD exceeded twice the average RSD of all laboratories).

To confirm those observations, *z*-scores were calculated for each individual amino acid result based on the average and SD of the 11 laboratories that did not show a global bias or extensive spread. An absolute *z*-score higher than 2 indicates that the result is more than 2 SDs away from the mean and is considered as questionable (9). As shown in Table 2, Laboratories 1, 5, and 13 had absolute *z*-scores values above 2 in 74%, 91%, and 46% of the cases, with average *z*-scores of 18.7, −5.7, and −2.1, respectively.

Laboratory 1 noted fluctuations of the norvaline peak in the standards and inconsistencies in sample peak areas. Laboratory 5 noted that the instrument had to be repaired during this period and that the injection volumes were too small. Nothing was reported from Laboratories 7 and 13.

In summary, data from Laboratories 1, 5, 7 and 13 showed constant and severe deviations and were considered as invalid. Similarly, tyrosine was systematically underestimated in Laboratory 2, and cysteine results were systematically underestimated in Laboratories 4 (SPIFAN powder and cereal samples) and 12 (all samples). All other values were considered as valid.

### Outlier Removal

For each analyte/sample pair, Cochran, Grubbs and double Grubbs statistical tests were conducted on the valid data using the “AOAC Interlaboratory Study Workbook—Blind (Unpaired) Replicates” template (https://www.aoac.org/resources) according to AOAC Appendix D (10; *see*Table 3 for details). No more than 2/9 laboratories were dropped for each analyte/sample pair during this process, and at least eight entries remained for each set except in a few cases, notably when the starting number of laboratories was reduced to nine or eight for the liquid samples (*see*Table 3 for details). Overall, 6% (164 out of 2857) of the values were judged outliers; about one third of the outliers were caused by a single laboratory, and more than 80% of outlier values were excluded after a Cochran test. Invalid data and outliers are highlighted in Supplemental File 1.

**Table 3.** Summary of precision statistics for studied samples for each amino acid (in alphabetical order)

Samples^a^	S1	S2	S3	S4	S5	S6	S7	S8	S9	D1	D2	D3	D4	C1	C2	C3
Alanine																

No. valid labs	11	11	11	11	9	11	11	11	11	9	10	10	10	10	10	10
No. outliers	0	1	1	1	0	0	1	0	0	0	0	0	0	0	0	0
Outlier type^b^	NA^c^	C^2^	C^2^	C^2^	NA	NA	C^15^	NA	NA	NA	NA	NA	NA	NA	NA	NA
No. duplicate values	22	20	20	20	18	22	20	22	22	18	20	20	20	20	20	20
Mean, mg/100g	59.96	66.53	59.92	60.88	59.73	49.45	60.87	59.70	86.74	107.76	516.11	2756.39	816.65	421.50	756.37	271.49
SD_r_, mg/100g^d^	1.41	0.79	1.04	0.86	0.96	0.52	0.92	1.14	1.28	2.68	13.58	38.63	16.56	4.77	19.24	5.59
SD_R_, mg/100g^e^	5.33	3.03	2.71	3.32	2.49	2.07	3.03	2.67	4.84	5.80	27.63	195.17	44.02	18.57	40.64	10.83
RSD_r_, %^f^	2.3	1.2	1.7	1.4	1.6	1.1	1.5	1.9	1.5	2.5	2.6	1.4	2.0	1.1	2.5	2.1
RSD_R_, %^g^	8.9	4.5	4.5	5.5	4.2	4.2	5.0	4.5	5.6	5.4	5.4	7.1	5.4	4.4	5.4	4.0
HorRat^h^	1.5	0.8	0.7	0.9	0.7	0.7	0.8	0.7	1.0	1.0	1.2	2.1	1.3	1.0	1.3	0.8

Arginine																

No. valid labs	11	11	11	11	9	11	11	11	11	9	10	10	10	10	10	10
No. outliers	0	1	1	1	3	0	0	0	2	1	0	0	2	0	1	0
Outlier type	NA	C^2^	C^2^	C^2^	DG^12 + 14^	NA	NA	NA	G^15^, C^9^	G^15^	NA	NA	C^14^, G^15^	NA	C^4^	NA
No. duplicate values	22	20	20	20	14	22	22	22	18	16	20	20	16	20	18	20
Mean, mg/100g	62.82	32.70	102.34	106.45	32.87	36.55	103.53	50.56	89.06	114.99	256.43	3448.93	850.69	463.27	839.06	254.22
SD_r_, mg/100g	2.30	0.87	1.88	2.14	1.47	1.12	2.18	0.89	2.42	4.33	18.88	61.37	18.23	9.72	37.47	6.98
SD_R_, mg/100g	5.16	1.54	4.93	4.07	1.84	2.08	4.74	2.10	3.59	9.81	18.88	202.11	32.56	32.82	48.41	20.83
RSD_r_, %	3.7	2.6	1.8	2.0	4.5	3.1	2.1	1.8	2.7	3.8	7.4	1.8	2.1	2.1	4.5	2.7
RSD_R_, %	8.2	4.7	4.8	3.8	5.6	5.7	4.6	4.2	4.0	8.5	7.4	5.9	3.8	7.1	5.8	8.2
HorRat	1.4	0.7	0.9	0.7	0.8	0.9	0.8	0.7	0.7	1.5	1.5	1.8	0.9	1.6	1.4	1.7

Aspartic acid																

No. valid labs	11	11	11	11	9	11	11	11	11	9	10	10	10	10	10	10
No. outliers	2	0	1	0	0	0	0	0	1	0	1	0	0	0	0	1
Outlier type	C^15^, C^2^	NA	C^2^	NA	NA	NA	NA	NA	C^9^	NA	C^4^	NA	NA	NA	NA	C^4^
No. duplicate values	18	22	20	22	18	22	22	22	20	18	18	20	20	20	20	18
Mean, mg/100g	143.13	150.73	163.78	181.99	20.50	112.79	166.81	139.04	137.17	259.28	1148.04	6380.08	1950.00	619.57	1791.73	374.41
SD_r_, mg/100g	1.24	4.24	4.09	9.01	0.47	1.42	4.57	2.62	2.45	7.02	24.04	115.91	61.82	9.06	54.62	10.02
SD_R_, mg/100g	6.95	6.29	6.44	9.24	1.42	4.69	7.81	5.29	7.59	23.18	55.50	550.09	90.37	30.98	132.46	21.60
RSD_r_, %	0.9	2.8	2.5	5.0	2.3	1.3	2.7	1.9	1.8	2.7	2.1	1.8	3.2	1.5	3.0	2.7
RSD_R_, %	4.9	4.2	3.9	5.1	6.9	4.2	4.7	3.8	5.5	8.9	4.8	8.6	4.6	5.0	7.4	5.8
HorRat	0.9	0.8	0.7	1.0	1.0	0.7	0.9	0.7	1.0	1.8	1.2	2.8	1.3	1.2	2.0	1.2

Cysteine/cystine																

No. valid labs	9	9	9	9	8	9	9	9	10	8	9	9	9	8	8	8
No. outliers	0	1	1	0	0	0	0	1	1	0	2	0	1	0	0	0
Outlier type	NA	C^2^	C^2^	NA	NA	NA	NA	C^14^	C^9^	NA	C^6^, C^4^	NA	G^11^	NA	NA	NA
No. duplicate values	18	16	16	18	16	18	18	16	18	16	14	18	16	16	16	16
Mean, mg/100g	18.12	35.11	17.96	24.44	136.86	19.61	18.01	20.89	16.68	25.87	258.89	389.14	199.79	177.19	187.80	125.64
SD_r_, mg/100g	0.98	0.59	0.49	1.41	6.51	0.42	0.82	0.26	0.30	0.73	4.43	9.97	7.01	5.61	7.36	2.89
SD_R_, mg/100g	2.48	4.00	1.99	2.92	13.71	2.31	2.23	1.36	2.59	1.83	17.01	42.39	7.68	17.38	10.95	10.83
RSD_r_, %	5.4	1.7	2.7	5.8	4.8	2.2	4.6	1.3	1.8	2.8	1.7	2.6	3.5	3.2	3.9	2.3
RSD_R_, %	13.7	11.4	11.1	11.9	10.0	11.8	12.4	6.5	15.5	7.1	6.6	10.9	3.8	9.8	5.8	8.6
HorRat	1.9	1.7	1.5	1.7	1.9	1.6	1.7	0.9	2.1	1.0	1.3	2.4	0.8	1.9	1.1	1.6

Glutamic acid																

No. valid labs	11	11	11	11	9	11	11	11	11	9	10	10	10	10	10	10
No. outliers	2	1	1	2	0	0	1	0	0	0	0	0	0	0	0	1
Outlier type	C^15^, C^2^	C^2^	C^2^	C^2^, C^3^	NA	NA	C^15^	NA	NA	NA	NA	NA	NA	NA	NA	C^4^
No. duplicate values	18	20	20	18	18	22	20	22	22	18	20	20	20	20	20	18
Mean, mg/100g	336.73	242.41	276.82	234.66	279.34	248.35	282.93	334.29	242.67	720.02	1949.57	20287.3	5389.82	2663.74	2313.90	2010.72
SD_r_, mg/100g	2.52	3.46	5.37	2.24	4.24	2.21	3.50	6.36	3.87	17.34	66.75	332.32	111.38	33.54	86.34	43.40
SD_R_, mg/100g	12.05	8.72	12.15	8.53	9.93	6.74	8.32	10.81	9.58	48.59	80.68	1848.19	244.60	132.45	134.37	103.71
RSD_r_, %	0.7	1.4	1.9	1.0	1.5	0.9	1.2	1.9	1.6	2.4	3.4	1.6	2.1	1.3	3.7	2.2
RSD_R_, %	3.6	3.6	4.4	3.6	3.6	2.7	2.9	3.2	3.9	6.7	4.1	9.1	4.5	5.0	5.8	5.2
HorRat	0.8	0.7	0.9	0.7	0.7	0.6	0.6	0.7	0.8	1.6	1.1	3.6	1.5	1.4	1.6	1.4

Glycine																

No. valid labs	11	11	11	11	9	11	11	11	11	9	10	10	10	10	10	10
No. outliers	2	1	1	0	2	0	0	0	2	1	0	0	0	0	1	0
Outlier type	C^15^, G^14^	C^2^	C^2^	NA	DG^12 + 14^	NA	NA	NA	C^9^, G^15^	G^12^	NA	NA	NA	NA	C^3^	NA
No. duplicate values	18	20	20	22	14	22	22	22	18	16	20	20	20	20	18	20
Mean, mg/100g	36.21	24.74	57.91	46.77	27.36	22.64	58.34	30.62	150.75	62.24	228.03	1745.01	471.33	495.74	789.29	312.98
SD_r_, mg/100g	0.58	0.31	1.23	1.52	0.65	0.35	1.34	0.68	2.24	2.68	13.09	43.78	14.49	7.63	7.95	7.94
SD_R_, mg/100g	2.97	1.48	2.57	2.68	1.12	1.24	3.23	1.71	9.03	3.27	17.33	116.89	21.19	30.32	23.72	17.32
RSD_r_, %	1.6	1.3	2.1	3.3	2.4	1.5	2.3	2.2	1.5	4.3	5.7	2.5	3.1	1.5	1.0	2.5
RSD_R_, %	8.2	6.0	4.4	5.7	4.1	5.5	5.5	5.6	6.0	5.3	7.6	6.7	4.5	6.1	3.0	5.5
HorRat	1.2	0.9	0.7	0.9	0.6	0.8	0.9	0.8	1.1	0.9	1.5	1.8	1.0	1.4	0.7	1.2

Histidine																

No. valid labs	11	11	11	11	9	11	11	11	11	9	10	10	10	10	10	10
No. outliers	2	2	0	2	2	0	1	2	1	2	0	0	0	0	1	1
Outlier type	C^15^, C^14^	C^15^, C^2^	NA	C^2^, C^15^	DG^12 + 14^	NA	C^15^	C^14^, C^11^	C^15^	C^12^, C^2^	NA	NA	NA	NA	C^3^	C^4^
No. duplicate values	18	18	22	18	14	22	20	18	20	14	20	20	20	20	18	18
Mean, mg/100g	40.32	24.04	35.06	44.01	24.82	27.19	34.80	40.25	43.85	88.47	187.18	2706.50	668.48	241.78	365.30	147.76
SD_r_, mg/100g	0.69	0.17	2.12	0.88	0.49	0.46	1.08	0.34	0.58	1.18	6.10	82.23	13.39	6.05	8.21	5.13
SD_R_, mg/100g	3.71	1.59	2.97	2.14	0.99	2.24	1.90	2.76	1.88	3.37	11.78	214.34	26.47	16.77	14.47	10.63
RSD_r_, %	1.7	0.7	6.0	2.0	2.0	1.7	3.1	0.8	1.3	1.3	3.3	3.0	2.0	2.5	2.2	3.5
RSD_R_, %	9.2	6.6	8.5	4.9	4.0	8.3	5.4	6.8	4.3	3.8	6.3	7.9	4.0	6.9	4.0	7.2
HorRat	1.4	0.9	1.3	0.8	0.6	1.2	0.8	1.1	0.7	0.7	1.2	2.3	0.9	1.4	0.9	1.3

Isoleucine																

No. valid labs	11	11	11	11	9	11	11	11	11	9	10	10	10	10	10	10
No. outliers	1	1	1	1	0	0	0	0	1	0	2	0	0	0	0	0
Outlier type	G^2^	C^2^	C^2^	C^2^	NA	NA	NA	NA	C^9^	NA	DG^2 + 9^	NA	NA	NA	NA	NA
No. duplicate values	20	20	20	20	18	22	22	22	20	18	16	20	20	20	20	20
Mean, mg/100g	86.01	84.03	66.78	120.87	83.28	69.61	68.11	88.45	87.11	175.95	670.64	4854.89	1296.03	352.38	657.58	254.06
SD_r_, mg/100g	0.88	1.44	1.68	1.48	1.17	1.01	1.92	1.65	0.60	3.85	18.91	60.69	18.71	7.24	20.67	7.78
SD_R_, mg/100g	3.37	3.51	3.13	4.14	4.51	2.73	2.50	3.20	2.50	12.89	18.91	276.04	71.31	22.66	34.42	19.96
RSD_r_, %	1.0	1.7	2.5	1.2	1.4	1.5	2.8	1.9	0.7	2.2	2.8	1.3	1.4	2.1	3.1	3.1
RSD_R_, %	3.9	4.2	4.7	3.4	5.4	3.9	3.7	3.6	2.9	7.3	2.8	5.7	5.5	6.4	5.2	7.9
HorRat	0.7	0.7	0.8	0.6	0.9	0.7	0.6	0.6	0.5	1.4	0.7	1.8	1.4	1.4	1.2	1.6

Leucine																

No. valid labs	11	11	11	11	9	11	11	11	11	9	10	10	10	10	10	10
No. outliers	1	1	2	1	1	0	0	0	1	1	0	0	0	0	0	1
Outlier type	C^2^	C^2^	C^2^, C^12^	C^2^	C^2^	NA	NA	NA	C^9^	C^2^	NA	NA	NA	NA	NA	C^4^
No. duplicate values	20	20	18	20	16	22	22	22	20	16	20	20	20	20	20	18
Mean, mg/100g	152.97	141.32	110.94	197.02	147.41	124.70	113.41	161.56	138.02	331.27	1104.06	8660.85	2424.44	672.95	1151.80	484.81
SD_r_, mg/100g	1.82	1.33	2.24	2.40	0.78	1.63	3.39	2.74	1.29	2.81	30.43	102.42	36.79	4.99	30.25	9.49
SD_R_, mg/100g	5.20	4.34	2.93	6.49	6.02	3.91	3.78	4.33	3.32	11.41	35.84	363.08	85.69	25.89	36.65	21.77
RSD_r_, %	1.2	0.9	2.0	1.2	0.5	1.3	3.0	1.7	0.9	0.8	2.8	1.2	1.5	0.7	2.6	2.0
RSD_R_, %	3.4	3.1	2.6	3.3	4.1	3.1	3.3	2.7	2.4	3.4	3.2	4.2	3.5	3.8	3.2	4.5
HorRat	0.6	0.6	0.5	0.6	0.8	0.6	0.6	0.5	0.4	0.7	0.8	1.5	1.0	0.9	0.8	1.0

Lysine																

No. valid labs	11	11	11	11	9	11	11	11	11	9	10	10	10	10	10	10
No. outliers	2	0	1	0	1	1	0	0	1	0	0	0	0	0	0	1
Outlier type	C^15^, G^2^	NA	C^2^	NA	C^3^	C^3^	NA	NA	C^9^	NA	NA	NA	NA	NA	NA	C^4^
No. duplicate values	18	22	20	22	16	20	22	22	20	18	20	20	20	20	20	18
Mean, mg/100g	131.43	122.87	86.15	110.16	97.40	99.47	84.17	130.72	91.06	260.13	959.10	7210.08	1952.96	190.94	813.25	99.54
SD_r_, mg/100g	2.70	3.04	2.12	4.84	1.64	1.39	2.32	4.08	1.88	12.14	39.87	188.35	54.39	6.04	29.69	5.43
SD_R_, mg/100g	10.86	4.50	3.82	5.17	9.18	3.99	3.65	5.61	4.82	22.89	42.58	564.60	104.64	14.07	35.04	9.55
RSD_r_, %	2.1	2.5	2.5	4.4	1.7	1.4	2.8	3.1	2.1	4.7	4.2	2.6	2.8	3.2	3.7	5.5
RSD_R_, %	8.3	3.7	4.4	4.7	9.4	4.0	4.3	4.3	5.3	8.8	4.4	7.8	5.4	7.4	4.3	9.6
HorRat	1.5	0.7	0.8	0.8	1.7	0.7	0.7	0.8	0.9	1.8	1.1	2.6	1.5	1.4	1.0	1.7

Methionine																

No. valid labs	11	11	11	11	9	11	11	11	11	9	10	10	10	10	10	10
No. outliers	2	0	1	1	2	0	0	0	1	0	0	0	0	0	2	1
Outlier type	C^15^, C^2^	NA	C^2^	C^2^	DG^12 + 14^	NA	NA	NA	C^9^	NA	NA	NA	NA	NA	DG^4 + 15^	C^4^
No. duplicate values	18	22	20	20	14	22	22	22	20	18	20	20	20	20	16	18
Mean, mg/100g	51.56	27.88	36.64	43.65	30.24	28.99	42.06	39.87	37.29	87.48	214.87	2655.25	643.26	136.08	138.29	101.89
SD_r_, mg/100g	0.59	1.13	0.88	1.02	0.99	0.60	0.42	0.70	0.40	4.05	10.38	57.73	18.39	2.87	8.20	3.32
SD_R_, mg/100g	3.02	2.03	1.68	1.94	1.77	1.22	1.81	1.96	1.65	7.51	24.80	157.39	41.25	11.42	26.48	7.19
RSD_r_, %	1.1	4.1	2.4	2.3	3.3	2.1	1.0	1.8	1.1	4.6	4.8	2.2	2.9	2.1	5.9	3.3
RSD_R_, %	5.9	7.3	4.6	4.5	5.9	4.2	4.3	4.9	4.4	8.6	11.5	5.9	6.4	8.4	19.1	7.1
HorRat	0.9	1.1	0.7	0.7	0.9	0.6	0.7	0.8	0.7	1.5	2.3	1.7	1.5	1.6	3.6	1.3

Phenylalanine																

No. valid labs	11	11	11	11	9	11	11	11	11	9	10	10	10	10	10	10
No. outliers	2	0	2	1	1	0	0	0	1	2	2	0	0	0	1	1
Outlier type	C^15^, C^2^	NA	C^2^, C^6^	C^2^	G^12^	NA	NA	NA	C^9^	C^12^, C^2^	C^6^, C^4^	NA	NA	NA	C^3^	C^4^
No. duplicate values	18	22	18	20	16	22	22	22	20	14	16	20	20	20	18	18
Mean, mg/100g	73.46	42.85	72.92	100.43	57.44	50.51	73.58	71.40	76.51	164.35	341.44	4856.93	1212.21	457.27	735.88	323.01
SD_r_, mg/100g	0.92	1.60	1.05	2.48	1.85	1.50	2.58	1.60	2.24	2.05	4.81	192.23	28.51	14.82	19.44	8.60
SD_R_, mg/100g	2.09	2.46	3.08	3.05	3.26	2.15	2.58	3.23	3.29	5.42	19.06	387.82	52.25	26.48	31.90	17.78
RSD_r_, %	1.3	3.7	1.4	2.5	3.2	3.0	3.5	2.2	2.9	1.2	1.4	4.0	2.4	3.2	2.6	2.7
RSD_R_, %	2.8	5.7	4.2	3.0	5.7	4.3	3.5	4.5	4.3	3.3	5.6	8.0	4.3	5.8	4.3	5.5
HorRat	0.5	0.9	0.7	0.5	0.9	0.7	0.6	0.8	0.7	0.6	1.2	2.5	1.1	1.3	1.0	1.2

Proline																

No. valid labs	11	11	11	11	9	11	11	11	11	9	10	10	10	10	10	10
No. outliers	1	1	1	1	0	0	0	1	1	1	1	0	0	0	0	0
Outlier type	C^2^	C^2^	C^2^	C^2^	NA	NA	NA	C^14^	C^9^	C^2^	C^6^	NA	NA	NA	NA	NA
No. duplicate values	20	20	20	20	18	22	22	20	20	16	18	20	20	20	20	20
Mean, mg/100g	136.23	79.14	71.80	60.39	113.78	100.43	73.35	140.65	128.49	334.02	635.36	9892.79	2404.13	858.97	902.26	671.88
SD_r_, mg/100g	1.52	0.97	1.15	1.38	1.99	0.90	1.69	1.42	2.34	2.58	11.07	112.57	37.75	10.41	27.87	19.89
SD_R_, mg/100g	4.45	2.85	1.50	2.27	5.23	3.12	2.39	4.14	3.92	12.74	22.07	466.92	87.63	28.02	29.87	35.65
RSD_r_, %	1.1	1.2	1.6	2.3	1.7	0.9	2.3	1.0	1.8	0.8	1.7	1.1	1.6	1.2	3.1	3.0
RSD_R_, %	3.3	3.6	2.1	3.8	4.6	3.1	3.3	2.9	3.1	3.8	3.5	4.7	3.6	3.3	3.3	5.3
HorRat	0.6	0.6	0.4	0.6	0.8	0.5	0.5	0.5	0.6	0.8	0.8	1.7	1.0	0.8	0.8	1.2

Serine																

No. valid labs	11	11	11	11	9	11	11	11	11	9	10	10	10	10	10	10
No. outliers	1	0	2	1	0	0	0	0	1	1	0	0	0	0	2	1
Outlier type	C^2^	NA	C^2^, G^12^	C^2^	NA	NA	NA	NA	C^9^	C^2^	NA	NA	NA	NA	DG^3 + 14^	C^4^
No. duplicate values	20	22	18	20	18	22	22	22	20	16	20	20	20	20	16	18
Mean, mg/100g	89.55	72.08	74.84	56.01	78.87	67.91	75.77	89.34	70.43	190.40	554.79	5378.17	1400.81	471.39	780.53	335.88
SD_r_, mg/100g	1.75	1.09	1.96	1.12	1.23	0.87	1.84	1.59	0.85	2.51	13.07	77.98	29.32	8.74	15.52	6.95
SD_R_, mg/100g	5.75	4.10	4.86	3.55	4.13	3.84	4.64	4.85	3.76	12.85	50.83	443.58	87.78	37.87	26.94	18.72
RSD_r_, %	2.0	1.5	2.6	2.0	1.6	1.3	2.4	1.8	1.2	1.3	2.4	1.4	2.1	1.9	2.0	2.1
RSD_R_, %	6.4	5.7	6.5	6.3	5.2	5.7	6.1	5.4	5.3	6.7	9.2	8.2	6.3	8.0	3.5	5.6
HorRat	1.1	1.0	1.1	1.0	0.9	0.9	1.0	0.9	0.9	1.3	2.1	2.7	1.6	1.8	0.8	1.2

Taurine																

No. valid labs	10	10	10	10	6	10	11	10	−^i^	−	−	−	−	−	−	−
No. outliers	2	1	1	2	1	0	1	1	−	−	−	−	−	−	−	−
Outlier type	C^15^, C^14^	C^15^	C^2^	C^2^, C^14^	C^14^	NA	G^10^	C^15^	−	−	−	−	−	−	−	−
No. duplicate values	16	18	18	16	10	20	20	18	−	−	−	−	−	−	−	−
Mean, mg/100g	3.78	4.23	4.62	3.45	4.76	4.38	5.93	3.04	−	−	−	−	−	−	−	−
SD_r_, mg/100g	0.14	0.33	0.17	0.18	0.47	0.25	0.38	0.20	−	−	−	−	−	−	−	−
SD_R_, mg/100g	1.07	0.68	0.80	0.66	0.64	0.78	0.86	0.67	−	−	−	−	−	−	−	−
RSD_r_, %	3.6	7.7	3.8	5.2	9.9	5.7	6.5	6.5	−	−	−	−	−	−	−	−
RSD_R_, %	28.2	16.0	17.3	19.1	13.4	17.9	14.4	21.9	−	−	−	−	−	−	−	−
HorRat	3.0	1.8	1.9	2.0	1.5	2.0	1.7	2.3	−	−	−	−	−	−	−	−

Threonine																

No. valid labs	11	11	11	11	9	11	11	11	11	9	10	10	10	10	10	10
No. outliers	2	1	1	2	0	0	1	0	1	1	1	0	0	0	1	0
Outlier type	C^2^, C^15^	C^2^	C^2^	C^2^, G^15^	NA	NA	C^15^	NA	C^9^	C^2^	C^6^	NA	NA	NA	G^3^	NA
No. duplicate values	18	20	20	18	18	22	20	22	20	16	18	20	20	20	18	20
Mean, mg/100g	76.89	95.08	53.68	77.72	86.13	67.55	55.10	81.80	68.88	150.85	719.29	4048.82	1099.23	319.87	611.55	217.19
SD_r_, mg/100g	0.54	0.82	0.79	2.00	1.48	0.62	0.73	1.41	0.70	1.54	12.35	55.32	21.25	4.93	11.64	5.07
SD_R_, mg/100g	4.03	3.72	2.43	3.65	3.99	2.89	2.32	3.18	2.77	6.09	31.31	245.13	45.53	12.03	18.09	9.31
RSD_r_, %	0.7	0.9	1.5	2.6	1.7	0.9	1.3	1.7	1.0	1.0	1.7	1.4	1.9	1.5	1.9	2.3
RSD_R_, %	5.2	3.9	4.5	4.7	4.6	4.3	4.2	3.9	4.0	4.0	4.4	6.1	4.1	3.8	3.0	4.3
HorRat	0.9	0.7	0.7	0.8	0.8	0.7	0.7	0.7	0.7	0.8	1.0	1.9	1.1	0.8	0.7	0.9

Tyrosine																

No. valid labs	10	10	10	10	8	10	10	10	10	8	9	9	9	9	9	9
No. outliers	2	1	1	1	1	1	0	0	1	1	0	0	0	0	1	2
Outlier type	C^15^, C^14^	C^14^	G^12^	C^15^	G^12^	C^3^	NA	NA	C^9^	G^12^	NA	NA	NA	NA	C^3^	C^4^, G^3^
No. duplicate values	16	18	18	18	14	18	20	20	18	14	18	18	18	18	16	14
Mean, mg/100g	72.76	38.01	51.71	92.30	53.74	50.11	53.04	73.06	66.95	172.16	289.45	5480.98	1229.56	272.80	478.83	192.23
SD_r_, mg/100g	0.63	0.43	1.85	2.94	2.04	0.88	2.33	1.56	1.90	1.43	21.81	195.15	32.86	11.67	10.51	5.19
SD_R_, mg/100g	3.70	2.11	2.45	4.42	2.78	3.08	3.20	3.12	2.86	6.07	30.59	384.91	70.98	34.42	23.51	7.32
RSD_r_, %	0.9	1.1	3.6	3.2	3.8	1.8	4.4	2.1	2.8	0.8	7.5	3.6	2.7	4.3	2.2	2.7
RSD_R_, %	5.1	5.5	4.7	4.8	5.2	6.2	6.0	4.3	4.3	3.5	10.6	7.0	5.8	12.6	4.9	3.8
HorRat	0.9	0.8	0.8	0.8	0.8	1.0	1.0	0.7	0.7	0.7	2.2	2.3	1.5	2.6	1.1	0.7

Valine																

No. valid labs	11	11	11	11	9	11	11	11	11	9	10	10	10	10	10	10
No. outliers	1	2	1	1	0	0	0	0	1	1	0	0	0	0	0	0
Outlier type	C^2^	C^2^, C^6^	C^6^	C^2^	NA	NA	NA	NA	C^9^	C^2^	NA	NA	NA	NA	NA	NA
No. duplicate values	20	18	20	20	18	22	22	22	20	16	20	20	20	20	20	20
Mean, mg/100g	95.93	77.65	66.94	139.19	87.20	75.12	69.28	99.18	99.86	212.44	617.05	6088.79	1555.51	468.02	769.70	325.74
SD_r_, mg/100g	0.66	0.50	1.66	1.20	1.24	0.80	2.06	1.68	0.92	2.11	14.59	52.65	20.49	8.23	22.93	7.49
SD_R_, mg/100g	4.44	3.16	3.56	5.61	4.40	2.38	2.88	3.63	3.40	10.89	31.08	342.90	88.39	20.67	31.93	17.54
RSD_r_, %	0.7	0.6	2.5	0.9	1.4	1.1	3.0	1.7	0.9	1.0	2.4	0.9	1.3	1.8	3.0	2.3
RSD_R_, %	4.6	4.1	5.3	4.0	5.0	3.2	4.2	3.7	3.4	5.1	5.0	5.6	5.7	4.4	4.1	5.4
HorRat	0.8	0.7	0.9	0.7	0.9	0.5	0.7	0.6	0.6	1.0	1.2	1.8	1.5	1.0	1.0	1.1

^a^S1: SRM 1869 (Infant/Adult Nutritional Formula II); S2: IF, partially hydrolysed, milk-based; S3: IF, partially hydrolysed, soy-based; S4: IF, elemental, amino-acid-based; S5: IF, ready-to-feed, milk-based; S6: IF, milk-based; S7: IF, soy-based; S8: toddler formula, milk-based; S9: adult nutritional powder, low-fat; D1: M-0142 (UHT skimmed milk); D2: MO-0614 (whey powder); D3: CA-0904 (sodium caseinate); D4: SRM 1549a (whole milk powder); C1: bran pet food; C2: dry pet food; C3: SRM 3233 (fortified breakfast cereal). Samples S5, D1, D2, D3, D4, C1, C2, and C3 are expressed as mg/100 g “as is”. Samples S1, S2, S3, S4, S6, S7, S8, and S9 are expressed as mg/100 g reconstituted final product. SRM 1869, 1549a, and 3233 are products from NIST. M-0142, MO-0614, and CA-0904 are products from muva kempten GmbH. This information is given for the convenience of users of this document and does not constitute an endorsement of the product named. Equivalent products may be used if they can be shown to lead to the same results.

^b^Outliers flagged by (C) Cochran, (G) Grubbs, or (DG) double Grubbs tests. The number(s) in superscript refer to the laboratories concerned. Two subsequent flagging events are separated by a comma. For arginine (sample S5), one laboratory reported only one value for one blind duplicate, and two laboratories were eliminated following a double Grubbs test.

^c^NA = Not applicable (no outlier was removed).

^d^Standard deviation of repeatability.

^e^Standard deviation of reproducibility.

^f^Relative standard deviation of repeatability. Values exceeding those listed in AOAC SMPR 2014.013 are underlined.

^g^Relative standard deviation of reproducibility. Values exceeding those listed in AOAC SMPR 2014.013 are underlined.

^h^Horwitz ratio.

^i^– = Not relevant.

### Performance Data

Final performance data were computed after outlier removal and are summarized in Table 3 (see Supplemental File 2 which lists performance data prior to outlier removal). Table 3 lists for each analyte/sample pair the number of valid results, outliers, and remaining duplicates, as well as the average value, SD of repeatability (SD_r_) and reproducibility (SD_R_), RSD of repeatability (RSD_r_) and reproducibility (RSD_R_), and Horwitz ratio (HorRat). The concentrations are expressed in mg per 100 g of reconstituted product for SPIFAN powder samples and in mg per 100 g of product (liquid or powder) for the other samples. Most samples have HorRat values that are between 0.5 and 2, which indicates “Method reproducibility as normally would be expected” (10).

In SPIFAN matrixes, RSD_r_ and RSD_R_ values were compared to the performance criteria defined in SMPR 2014.013 (8), and exceeding values are underlined in Table 3. Repeatability requirements were met for 154 of the 161 analyte/sample pairs analyzed. Reproducibility requirements were met for 135 of the 161 analyte/sample pairs analyzed; exceeding values were mostly observed for cysteine and taurine, with average RSD_R_ values of 11.6% and 18.5%, respectively. For all other amino acids, the RSD_R_ values were within the SMPR boundaries in 134 out of 144 cases, and only two values were above the RSD_r_ threshold.

Cystine calibrants must be derivatized prior to injection, which most likely increases the variability for this analyte. However, being able to analyze sulfur amino acids at the same time as the other amino acids represents an important improvement over existing methods and the SPIFAN Nutrients Expert Review Panel recommended keeping cysteine in the final method.

Taurine levels are about 10–100 times lower than those of the other amino acids, leading to higher RSD_R_ values. The SPIFAN Nutrients Expert Review Panel recommended the exclusion of taurine from the final method because other methods such as AOAC Method **997.05** (11) can measure low levels of taurine with higher accuracy. Taurine results are included in this manuscript for the sake of completeness, and readers can refer to the single-laboratory validation manuscript (7) for the taurine protocol.

In dairy and cereal matrixes, 78 of the 111 analyte/sample observations had an RSD_r_ below 3%, 28 observations were between 3 and 5%, and 5 were above 5%. For RSD_R_, 38 of the 111 observations were below 5%, 54 between 5 and 8%, and 19 above 8%.

Several laboratories used at least one in-house alternative reagent. Laboratory 6 used only in-house alternative reagents and provided accurate results, with 84% of *z*-scores between −1 and 1 (228/273), no *z*-score above 2, and an average *z*-score of −0.01 (see Supplemental Table 2).

Recovery had previously been assessed by spike experiments during the single-laboratory validation (7). During this interlaboratory study, trueness was assessed by comparing the average values calculated in sample S1 (NIST SRM 1869), D4 (NIST SRM 1549a), and C3 (NIST SRM 3233) with the reference values from the corresponding certificate of analysis (CoA). As shown in Table 4, 37/50 amino acid values were within the concentration range described in the CoA, and 39/50 values were within 10% of the reference values. The differences were larger for the cereal reference sample C3. This might be specific to this sample and not to the matrix type since values for samples C1 and C2 were within 10% from those obtained by a classical post-column derivatization method (Table 4; this comparison is purely informative since samples C1 and C2 were only analyzed once by the post-column derivatization method).

**Table 4.** Trueness evaluation by comparison with reference values from standard reference material (SRM) from NIST as well as parallel quantification using post-column derivatization in an amino acid analyzer (AAA)^a^

	S1 (NIST SRM 1869)					D4 (NIST SRM 1549a)					
	Ref.	Ref^c^_Min__._	Ref_Max__._	Interlaboratory	% of Ref.	Ref.	Ref_Min._	Ref_Max._	Interlaboratory	% of Ref.	
Alanine	539.0	514.0	564.0	539.7	100	845.0	761.0	929.0	816.7	97	
Arginine	571.0	527.0	615.0	565.3	99	890.0	750.0	1030.0	850.7	96	
Asprartic acid	1300.0	1283.0	1317.0	1288.1	99	1960.0	1900.0	2020.0	1950.0	99	
Cystine	149.0	140.2	157.8	163.1	109	180.0	160.0	200.0	199.8	111	
Glutamic acid	2936.0	2855.0	3017.0	3030.6	103	5340.0	5120.0	5560.0	5389.8	101	
Glycine	325.1	320.2	330.0	325.9	100	460.0	420.0	500.0	471.3	102	
Histidine	365.1	355.3	374.9	362.9	99	617.0	534.0	700.0	668.5	108	
Isoleucine	778.0	764.0	792.0	774.1	100	1120.0	1100.0	1140.0	1296.0	116	
Leucine	1394.0	1371.0	1417.0	1376.7	99	2410.0	2385.0	2435.0	2424.4	101	
Lysine	1184.0	1145.0	1223.0	1182.9	100	2050.0	2038.0	2062.0	1953.0	95	
Methionine	474.0	435.0	513.0	464.1	98	680.0	670.0	690.0	643.3	95	
Phenylalanine	682.3	675.2	689.4	661.2	97	1210.0	1100.0	1320.0	1212.2	100	
Proline	1260.0	1219.0	1301.0	1226.1	97	NA^b^	NA	NA	2404.1	NA	
Serine	805.0	771.0	839.0	806.0	100	1420.0	1400.0	1440.0	1400.8	99	
Taurine	37.2	34.0	40.4	34.0	91	NA	NA	NA	NA	NA	
Threonine	696.0	683.0	709.0	692.0	99	1090.0	1030.0	1150.0	1099.2	101	
Tyrosine	610.0	528.0	692.0	654.9	107	1120.0	1060.0	1180.0	1229.6	110	
Valine	861.0	831.0	891.0	863.4	100	1340.0	1080.0	1600.0	1555.5	116	

	C3 (NIST SRM 3233)					C1			C2		
			
	Ref	Ref_Min._	Ref_Max._	Interlaboratory	% of Ref	AAA	Interlaboratory	% of AAA	AAA	Interlaboratory	% of AAA

Alanine	317.5	276.2	358.8	271.5	86	440.0	421.5	96	790.0	756.4	96
Arginine	316.5	250.7	382.4	254.2	80	470.0	463.3	99	890.0	839.1	94
Asprartic acid	430.6	381.4	479.7	374.4	87	630.0	619.6	98	1820.0	1791.7	98
Cystine	151.4	119.9	182.8	125.6	83	222.0	177.2	80	252.0	187.8	75
Glutamic acid	2211.8	1995.5	2428.0	2010.7	91	2940.0	2663.7	91	2540.0	2313.9	91
Glycine	336.2	305.7	366.7	313.0	93	490.0	495.7	101	800.0	789.3	99
Histidine	159.2	125.8	192.7	147.8	93	240.0	241.8	101	380.0	365.3	96
Isoleucine	265.4	251.6	279.2	254.1	96	350.0	352.4	101	660.0	657.6	100
Leucine	540.7	494.4	586.9	484.8	90	670.0	672.9	100	1160.0	1151.8	99
Lysine	101.2	61.9	140.6	99.5	98	170.0	190.9	112	800.0	813.2	102
Methionine	136.6	118.0	155.3	101.9	75	143.0	136.1	95	308.0	138.3	45
Phenylalanine	366.7	334.2	399.1	323.0	88	470.0	457.3	97	770.0	735.9	96
Proline	NA	NA	NA	671.9	NA	880.0	859.0	98	920.0	902.3	98
Serine	368.6	307.7	429.6	335.9	91	460.0	471.4	102	770.0	780.5	101
Threonine	236.9	224.1	249.7	217.2	92	320.0	319.9	100	630.0	611.6	97
Tyrosine	227.1	170.1	284.1	192.2	85	240.0	272.8	114	450.0	478.8	106
Valine	337.2	311.6	362.7	325.7	97	520.0	468.0	90	860.0	769.7	90

a Results outside of the reference values are underlined.

b NA = Not applicable.

c Ref_Min_ and Ref_Max_ = Minimal and Maximal reference values considering an expanded uncertainty at 95% confidence level.

## Conclusions

An interlaboratory study for method AOAC Method **2018.06** was successfully conducted in sixteen SPIFAN, dairy, and cereal matrixes by 15 different laboratories using five different UHPLC instruments. The laboratories used either commercial or in-house reagents, demonstrating that the method is not limited to a single supplier.

This interlaboratory study demonstrates the robustness and fitness-for-purpose of this method in different matrix groups, and the method is expected to be applicable to a wider variety of matrixes based on its harsh but robust chemistry. This study can now serve as a benchmark for future method improvements such as microwave-assisted hydrolysis and detection using single or triple quadrupole mass spectrometers.

The initial data validation step was questioned by the AOAC SPIFAN Nutrient Expert Review Panel, IDF Standing Committee on Analytical Methods for Composition (SCAMC), and IDF Standing Committee on Statistics and Automation (SCSA). The main argument against the rejection of laboratories showing too much bias or spread was that the method performance might be artificially overestimated, and that it will be difficult for new laboratories to match those performances when implementing the method. We are confident that the method performances presented here are realistic and that the invalid data do not represent the true performance of the laboratories concerned (two of them reported technical issues, and two of them did not return results from the practice samples). New laboratories that will implement this method will have time to properly verify their performance using standard reference materials such as those described in this study.

## Supplementary Material

qsac083_Supplementary_Data
