# Supplementary material for: Determination of Total Amino Acids in Infant Formulas, Adult Nutritionals, Dairy, and Cereal Matrixes by UHPLC–UV: Interlaboratory Validation Study, Final Action 2018.06
Source: J AOAC Int. 2022 Jun 29;105(6):1625–39. doi: 10.1093/jaoacint/qsac083 (PMC9605775; doi:10.1093/jaoacint/qsac083)
Supplement: qsac083_Supplementary_Data [file qsac083_supplementary_data.zip › qsac083_Supplementary_Data/aoac-22-0019-File003.docx]

Supplementary Table 1: Equipment and chemicals used by the different laboratories. AccQ·Tag refers to chemicals from Waters (AccQ·Tag Ultra Eluent A, Eluent B, and Derivatization kit). DNR: Did Not Reply.

|  | UHPLC apparatus | Mobile phase A | Mobile phase B | Derivatizing Reagents | Comments from the participating laboratory |
| --- | --- | --- | --- | --- | --- |
| Laboratory 1 | Waters Acquity (binary) | AccQ·Tag | AccQ·Tag | AccQ·Tag | Ampules were used for sample hydrolysis rather than vials |
| Laboratory 2 | Thermo 3000 RS | AccQ·Tag | Alternative | AccQ·Tag |  |
| Laboratory 3 | DNR | DNR | DNR | DNR |  |
| Laboratory 4 | Waters Acquity H-Class | AccQ·Tag | AccQ·Tag | AccQ·Tag |  |
| Laboratory 5 | Waters Acquity | AccQ·Tag | Alternative | AccQ·Tag |  |
| Laboratory 6 | Waters Acquity (binary) | Alternative | Alternative | Alternative |  |
| Laboratory 7 | Waters Acquity H-Class (quaternary) | AccQ·Tag | AccQ·Tag | AccQ·Tag |  |
| Laboratory 8 | Agilent 1260 Infinity II | AccQ·Tag | Alternative | AccQ·Tag | Minor changes done on gradient to achieve satisfactory separation on our instrument |
| Laboratory 9 | Waters Acquity (binary) | AccQ·Tag | Alternative | AccQ·Tag |  |
| Laboratory 10 | Waters Acquity I-Class (binary) | AccQ·Tag | AccQ·Tag | AccQ·Tag | 40 μL water were added to each sample and standard after tagging to mitigate the impact of sample fronting on early eluting analytes |
| Laboratory 11 | Waters Acquity | AccQ·Tag | AccQ·Tag | AccQ·Tag |  |
| Laboratory 12 | Waters Acquity H-Class | AccQ·Tag | AccQ·Tag | AccQ·Tag | 120 mL of the Eluent A extract in 1 liter (instead of 150 mL) for better separation between mono-lys and taurine |
| Laboratory 13 | DNR | DNR | DNR | DNR |  |
| Laboratory 14 | Waters Acquity H-Class | AccQ·Tag | Alternative | AccQ·Tag |  |
| Laboratory 15 | Waters Acquity | AccQ·Tag | AccQ·Tag | AccQ·Tag |  |

Supplementary Table 2: *z*-scores obtained by laboratory 6 that used only in-house reagents for mobile phases A and B and derivatizing reagents. C: outlier values according to the Cochran test.

|  | **S1** | **S2** | **S3** | **S4** | **S5** | **S6** | **S7** | **S8** | **S9** | **D1** | **D2** | **D3** | **D4** | **C1** | **C2** | **C3** |
| --- | --- | --- | --- | --- | --- | --- | --- | --- | --- | --- | --- | --- | --- | --- | --- | --- |
| Alanine | 0.4 | 0.9 | 0.6 | 0.8 | 1.1 | 0.8 | 1.1 | 0.9 | 0.6 | 1.1 | 1.3 | 0.7 | 0.3 | 1.1 | 0.6 | 1.6 |
| Arginine | -0.8 | -1.3 | -0.3 | -0.9 | -0.2 | -0.3 | 0.0 | -0.6 | -0.4 | -0.2 | 0.8 | -0.1 | -1.0 | -0.6 | -0.2 | 0.4 |
| Asprartic acid | 1.6 | 1.6 | 0.6 | 0.1 | 1.0 | 1.7 | 1.8 | 2.0 | 1.2 | 0.8 | 1.7 | 1.0 | 0.2 | 1.8 | 0.6 | 1.6 |
| Cystine | -0.7 | -0.7 | -0.4 | -0.2 | 0.1 | -1.0 | -0.9 | -1.4 | -0.8 | 1.7 | C | 0.7 | 1.3 | -0.9 | -0.3 | -1.4 |
| Glutamic acid | 0.5 | 0.6 | -0.1 | 0.6 | 0.9 | 0.6 | 1.0 | 0.7 | 0.5 | 1.0 | 0.8 | 0.5 | 0.1 | 0.3 | 0.4 | 0.9 |
| Glycine | -0.5 | 0.0 | 0.3 | -0.3 | 0.1 | 0.3 | 0.5 | 0.0 | -0.1 | 0.2 | 0.6 | -0.5 | -0.5 | -1.0 | -1.0 | -0.4 |
| Histidine | -0.8 | -1.0 | -0.3 | -0.4 | -0.8 | -0.2 | -0.1 | -0.8 | -0.2 | -0.4 | C | -0.2 | -0.9 | -0.8 | -0.7 | 0.0 |
| Isoleucine | -0.9 | -1.6 | -1.3 | -0.6 | -0.6 | -0.8 | -0.4 | -0.9 | -1.0 | -0.5 | -0.2 | -0.1 | -0.9 | -0.8 | -0.1 | 0.2 |
| Leucine | -1.1 | -1.5 | -0.8 | -0.9 | -1.1 | -0.9 | -0.5 | -1.0 | -0.9 | -1.0 | 0.3 | -0.8 | -1.5 | -0.6 | 0.0 | 0.2 |
| Lysine | 0.2 | 0.7 | -0.3 | 0.6 | 0.7 | 0.9 | 1.4 | 1.1 | 0.3 | 0.8 | 0.7 | 0.7 | 0.3 | 1.5 | 0.7 | 1.4 |
| Methionine | -0.3 | -0.4 | 0.0 | -0.5 | 0.4 | 0.8 | 0.4 | 0.2 | 0.6 | 0.2 | 0.7 | -0.2 | -0.3 | 0.1 | 0.7 | 0.6 |
| Phenylalanine | -1.0 | -0.7 | C | -0.8 | -0.6 | 0.0 | 0.0 | -0.3 | -0.1 | -0.2 | C | -0.5 | -0.8 | -0.9 | -0.5 | -0.3 |
| Proline | -0.7 | -0.7 | 0.1 | -1.1 | -0.6 | -0.3 | 0.2 | -0.6 | -0.3 | -0.7 | C | -0.7 | -1.4 | -0.2 | 0.1 | 0.0 |
| Serine | -0.3 | -0.3 | -0.1 | 0.2 | 1.4 | 0.1 | 0.5 | -0.1 | 0.4 | 0.6 | 1.9 | -0.6 | 0.2 | 0.5 | 0.1 | 0.2 |
| Taurine | 0.2 | 0.5 | 0.8 | 0.8 | 0.4 | 0.4 | 0.8 | 0.9 |  |  |  |  |  |  |  |  |
| Threonine | -0.5 | -0.7 | 0.0 | 0.0 | -0.8 | 0.0 | 0.2 | -0.1 | -0.1 | -0.2 | C | -0.6 | -0.8 | -0.2 | -0.3 | 0.1 |
| Tyrosine | -0.9 | -0.8 | 0.1 | 0.4 | -1.4 | -0.1 | 0.0 | -0.4 | 0.2 | 0.0 | 1.6 | -0.6 | -0.1 | -0.2 | 0.5 | 0.7 |
| Valine | -0.6 | C | -1.2 | -0.4 | -0.4 | -1.1 | -0.6 | -1.0 | -0.8 | -0.7 | 0.3 | -0.4 | -0.8 | -0.8 | -0.3 | 0.4 |
